# Endemicity of *Pseudomonas aeruginosa* producing IMP-18 and/or VIM-2 MBLs from the high-risk clone ST111 in Central America

**DOI:** 10.1093/jacamr/dlad092

**Published:** 2023-08-01

**Authors:** Lalitagauri M Deshpande, Silvio Vega, Juan Carlos Tinoco, Mariana Castanheira

**Affiliations:** JMI Laboratories, North Liberty, IA 52317, USA; Complejo Hospitalario Metropolitano, Senacyt, Panamá; Hospital General 450, Secretaría de Salud, Durango, México; JMI Laboratories, North Liberty, IA 52317, USA

## Abstract

**Background:**

*Pseudomonas aeruginosa* is an important cause of serious nosocomial infections. Despite the overall genetic diversity of this species, highly conserved clonal complexes (CCs) have been observed among MDR isolates. Many of these CCs are associated with MBL-producing isolates.

**Objectives:**

To evaluate five *P. aeruginosa* isolates from Central America that carried IMP-18- and/or VIM-2-encoding genes from the SENTRY Antimicrobial Surveillance Program (2017–2018).

**Methods:**

Susceptibility testing was performed by broth microdilution (CLSI). WGS was performed using MiSeq (Illumina) and MinION (Oxford Nanopore). Assembled contigs from short and long reads were combined for *in silico* screening of resistance genes, MLST, core genome (cg)MLST and SNP analysis.

**Results:**

The *P. aeruginosa* isolates were collected in Panama and Mexico from patients with urinary tract infections or pneumonia. Isolates were categorized as XDR (CLSI/EUCAST). All isolates belonged to ST111 but carried different combinations of resistance-encoding genes. Transposon-associated MBL genes, *bla*_IMP-18_ and/or *bla*_VIM-2_, were chromosomally located. *bla*_IMP-18_ was detected in an In*1666* integron whereas *bla*_VIM-2_ was embedded in an In*59*-like integron. Isolates were closely related based on cgMLST (average allele distance 2–34) and SNP analysis (5–423 different SNPs).

**Conclusions:**

MBL-producing ST111 *P. aeruginosa* have become endemic in Panama and may have spread to Mexico via clonal dissemination. Recombination events are apparent in the evolution of this CC. Surveillance is warranted to track the expansion and movement of this clone.

## Introduction

*Pseudomonas aeruginosa* is an opportunistic pathogen responsible for a variety of invasive human infections, including serious nosocomial infections. MDR *P. aeruginosa* is classified as a serious threat by the CDC in the 2019 AR Threats Report^1^ and was ranked among the top five pathogens causing serious infections during the initial 20 years (1997–2016) of the global SENTRY Antimicrobial Surveillance Program.^2^ In this surveyed period, the prevalence of *P. aeruginosa* in the SENTRY Program was reported to be between 4.8% among bloodstream infections to as high as 18.0% among patients hospitalized with pneumonia. Additionally, a high percentage of *P. aeruginosa* isolated from patients with pneumonia (27.7% and 19.0%) and bloodstream infections (23.7% and 17.4%) exhibited MDR and XDR phenotypes, respectively.^2^

*P. aeruginosa* isolates display a non-clonal epidemic population structure. This population is defined by a superficially clonal structure with frequent recombinations in which highly successful epidemic clones occasionally emerge and disseminate.^3–5^ ST235, ST111 and ST233 are 3 of the 10 most prevalent *P. aeruginosa* high-risk clones. These clones are important due not only to their prevalence, but also to their global spread, MDR/XDR profiles, and their association with ESBLs and carbapenemases.^6^ Notably, ST111 and ST233 are MDR/XDR clones that have disseminated worldwide and are particularly linked to the VIM-2 MBL.^6^*P. aeruginosa* that produce IMP-18 are sporadically reported in the USA and Mexico,^7^ whereas isolates producing both IMP-18 and VIM-2 have been reported in Costa Rica in 2004 and 2005.^8^ This study evaluated five *P. aeruginosa* isolates that were submitted to the SENTRY Program from medical centres located in Central America and carried IMP-18- and/or VIM-2-encoding genes.

## Materials and methods

*P. aeruginosa* isolates collected during 2017–2018 as part of the SENTRY Program were tested for susceptibility by reference broth microdilution as described by the CLSI (M07, 2018).^9,10^ Quality control was performed according to the CLSI M100 (2023) guidelines.^9,10^

Five isolates identified as XDR (CLSI/EUCAST criteria) were further investigated.^11^ WGS was conducted using MiSeq (Illumina, San Diego, CA, USA) and MinION (Oxford Nanopore Technologies, Oxford, UK). DNA libraries for short-read sequencing were prepared using the Nextera XT^™^ library construction protocol and index kit (Illumina) and sequenced on a MiSeq Sequencer using MiSeq Reagent Kit v3 (600 cycle). Additionally, high molecular weight DNA was extracted using Nanobind CBB Big DNA Kit (Circulomics, Inc., Baltimore, MD, USA) to generate barcoded sequencing libraries using the Rapid Barcoding Kit (Oxford Nanopore). Long-read sequencing reactions were performed using a FLO-MIN106 flow cell (Oxford Nanopore) on the MinION Mk1C sequencing platform. Assembled contigs from short and long reads were combined for *in silico* screening of resistance genes using proprietary pipeline and MLST (https://github.com/tseemann/mlst). Core genome (cg)MLST (comparing 3937 core genes) and SNP analyses (using exclusion parameter of 0 and 10 nucleotides) were performed on the 1928 Diagnostics platform (https://www.1928diagnostics.com/). Isolates described in this study were compared at a nucleotide level with the ST111 *P. aeruginosa* AG1 (PaeAG1; GenBank accession number NZ_CP045739), which carried *bla*_IMP-18_ and *bla*_VIM-2_ and was isolated from a Costa Rican hospital in 2010.^12^

## Results

Four XDR *P. aeruginosa* isolates were collected from a medical centre in Panama whereas one XDR *P. aeruginosa* isolate was collected in Mexico. Isolates were non-susceptible to all β-lactam antimicrobials tested, including imipenem (MIC >8 mg/L), meropenem (>32 mg/L), aztreonam (≥16 mg/L), piperacillin/tazobactam (32–128 mg/L), ceftolozane/tazobactam (>32 mg/L) and ceftazidime/avibactam (≥32 mg/L). Isolates were also resistant to tobramycin (>16 mg/L), amikacin (four of five; >32 mg/L) and levofloxacin (>16 mg/L) (Table S1, available as Supplementary data at *JAC-AMR* Online). Colistin MIC values for these isolates (1 or 2 mg/L) were categorized as intermediate per CLSI breakpoints.^10^

All isolates belonged to ST111 but carried different combinations of resistance-encoding genes, including MBLs and aminoglycoside-modifying enzyme-encoding genes (Table 1). Interestingly, the isolates cultured from patients hospitalized with pneumonia (PA-1, PA-2 and PA-5) carried *bla*_IMP-18_ and *bla*_VIM-2_, whereas the isolates recovered from patients with UTIs (PA-3 and PA-4) carried either *bla*_IMP-18_ or *bla*_VIM-2_. These MBL genes were chromosomally located on separate integrons that were similar to those described in PaeAG1 from Costa Rica that carried VIM-2- and IMP-18-encoding genes.^12^ The *bla*_IMP-18_-carrying integron, In*1666*, also harboured *bla*_OXA-2_ and *aac(6′)-Ib* in all but one isolate, PA-5, which did not carry *aac(6′)-Ib*. *bla*_VIM-2_ was located on a separate In*59*-like integron that also carried *aac(6′)-29a* and *aac(6′)-29b*. The *aph(3′)-IIb*-like gene that was detected in all five isolates was not a part of the MBL-carrying integrons.

**Table 1.** Summary of demographic and molecular findings of ST111 *P. aeruginosa* isolates

Characteristic	PA-1	PA-2	PA-3	PA-4	PA-5
Country (Month-year isolated)	Panama (MAR-2017)	Panama (JUN-2017)	Panama (JUL-2017)	Mexico (JUL-2017)	Panama (MAR-2018)
Infection type	Pneumonia in hospitalized patients	Pneumonia in hospitalized patients	Urinary tract infection	Urinary tract infection	Pneumonia in hospitalized patients
Infection origin	Nosocomial	Community-acquired	Nosocomial	Community-acquired	Not known
β-Lactamase-encoding genes	***bla*_IMP-18_**, *bla*_OXA-2_, ***bla*_VIM-2_**	***bla*_IMP-18_**, *bla*_OXA-2_, ***bla*_VIM-2_**	***bla*_IMP-18_**, *bla*_OXA-2_	***bla*_VIM-2_**	***bla*_IMP-18_**, *bla*_OXA-2_, ***bla*_VIM-2_**
Aminoglycoside-modifying enzyme-encoding genes	*aac(6′)-29a, aac(6′)-29b, aac(6′)-Ib*, *aph(3′)-IIb*-like	*aac(6′)-29a, aac(6′)-29b, aac(6′)-Ib, aph(3′)-IIb*-like	*aac(6′)-29b, aac(6′)-Ib, aph(3′)-IIb*-like	*aac(6′)-29a*, *aac(6′)-29b, aph(3′)-IIb-*like	*aac(6′)-29a*, *aac(6′)-29b*, *aph(3′)-IIb*-like
cgMLST allele distance^a^	2	2	10	34	19
Raw SNP differences compared with PaeAG1 from Costa Rica^b^	336	331	283	1124	101

**Figure 1.**
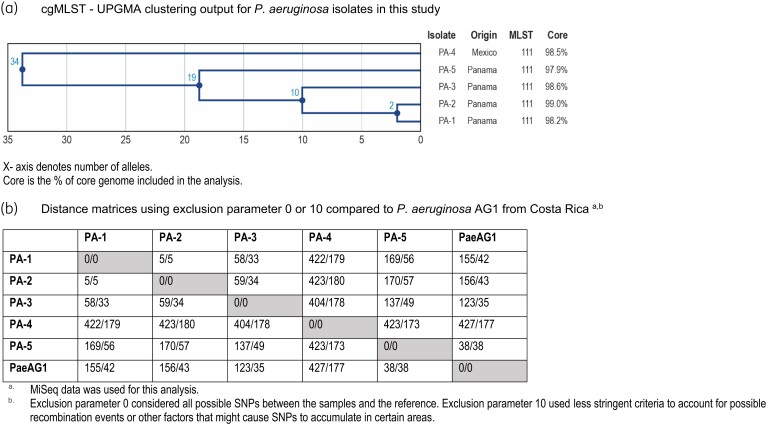
Summary of cgMLST and SNP analysis performed on the 1928 Diagnostics platform. (a) cgMLST—Unweighted Pair Group Method with Arithmetic Mean (UPGMA) clustering output for *P. aeruginosa* isolates in this study. The *x*-axis denotes the number of alleles. Core is the percentage of core genome included in the analysis. (b) Distance matrices using exclusion parameter 0 or 10 compared with *P. aeruginosa* AG1 from Costa Rica. MiSeq data were used for this analysis. Exclusion parameter 0 considered all possible SNPs between the samples and the reference. Exclusion parameter 10 used less stringent criteria to account for possible recombination events or other factors that might cause SNPs to accumulate in certain areas.

MBL encoding genes are printed in bold type.MBL encoding genes are printed in bold type.

a Depicted in Figure 1(a).

b Analysis was performed on the 1928 Diagnostics bioinformatic platform. The consensus sequence that combined short and long reads from each sample was individually compared with the reference sequence.

cgMLST results confirmed that these five isolates were closely related, with 2 to 19 allele differences among the four isolates from Panama and 34 allele differences in the one isolate from Mexico (Table 1 and Figure 1a). Between 101 and 1124 raw SNP differences were observed when the consensus sequence for each isolate, generated by combining short reads and long reads, was individually compared with the published reference sequence of PaeAG1 (CP045739.1) (Table 1).

A SNP analysis using only short-read data that considered all SNPs between sample to sample and sample to reference (exclusion parameter 0, Figure 1b) demonstrated that there were 5 to 423 nt differences among the five isolates evaluated in this study. The two isolates from Panama that were collected in 2017 (PA-1 and PA-2) were closely related to each other and displayed only 5 nt differences. Isolate PA-3 from Panama, which was also collected in 2017, exhibited 58 or 59 nt differences when compared with the isolates from the same year, but more divergence (137–170 nt) compared with isolate PA-5 collected in Panama a year later. The isolate collected in Mexico exhibited the most genetic distance, as it displayed 404 to 423 nt differences when compared with the four isolates from Panama. Isolate PA-5 collected in Panama in 2018 was most similar to PaeAG1, as it had 38 SNPs, whereas isolate PA-4 from Mexico showed the most SNP variations (427 nt differences). When the SNP exclusion parameter of 10 was applied, which excluded all SNPs within 10 nt to one another, the isolates from Panama and Mexico showed between 5 and 180 SNP differences (Figure 1b). Compared with the PaeAG1, these isolates exhibited 35 to 177 SNPs. This less stringent SNP analysis accounted for possible recombination events or other factors that might cause SNPs to accumulate in certain genomic regions.

## Discussion

*P. aeruginosa* isolates inhabit highly selective environments that lead to the development of intrinsic resistance mechanisms as well as the acquisition of resistance markers. Accordingly, *P. aeruginosa* isolates become potential reservoirs of resistance genes, and their genetic plasticity allows these bacteria to transfer a wide variety of acquired resistance genes. Integrons carrying MBLs and other resistance genes in *P. aeruginosa* are generally located on chromosomal genomic islands or transposable elements,^13^ making them resilient to loss under non-selective conditions. These traits also make the transfer of resistance determinants more efficient. In this study, the isolates from patients with invasive hospital-acquired pneumonia carried two MBL genes whereas isolates from UTI patients each carried just one MBL gene, all on chromosomally located transposons.

*P. aeruginosa* isolates carrying multiple MBLs have been reported from many parts of the world including India (IMP, VIM and NDM types with a combination of two types or all three types in one isolate),^14^ Greece (IMP and VIM types)^15^ and Costa Rica (IMP and VIM types).^8^ Specifically, the report from Costa Rica was of an outbreak of *P. aeruginosa* isolates producing IMP-18 and VIM-2 in 2004–2005. Of these isolates, 88.9% belonged to diverse clonal types based on ERIC-PCR analysis, suggesting that the *bla*_IMP_ and *bla*_VIM_ genes detected were probably linked in a genetic structure susceptible to lateral transfer.^8^ A representative ST111 isolate, PaeAG1 from the Costa Rican outbreak, was later characterized.^12^ This isolate carried *bla*_IMP-18_ and *bla*_VIM-2_ and harboured 57 genomic islands. Because of the characteristics, which were similar to the isolates reported in this study, PaeAG1 was used as an index strain for these genetic comparisons. Analysis on the 1928 Diagnostics platform revealed differences among these ST111 isolates. cgMLST analysis mapped the Panama isolates as evolutionarily closer to one another (2–19 allele differences) than to the isolate from Mexico (34 alleles difference). Notably, among the isolates from Panama, isolate PA-5 from March 2018 showed the least SNP differences from PaeAG1. The isolate from Mexico was the most distinct from PaeAG1 as well as the isolates from Panama, suggesting that the Costa Rican isolates may have disseminated and become endemic in Panama.

Overall, these analyses revealed genetic variability among apparently clonal outbreak isolates originating from community- and hospital-acquired infections. Isolates from this study were recovered in a close time frame; four were from the same medical centre. Despite temporal proximity, the differences observed in the SNP analysis and MBL gene variations are indicative of recombination events that play a role in the evolution of this clonal complex in this region. The MDR nature of these isolates highlights the need for availability of more effective treatment options in this region. Surveillance is warranted to track the expansion and movement of this clone, as these two MBL genes are associated.

## Supplementary Material

dlad092_Supplementary_Data

## Supplementary data

Table S1 is available as Supplementary data at *JAC-AMR* Online.

## References

[1] CDC . Antibiotic Resistance Threats in the United States, 2019. Centers for Disease Control and Prevention, 2019.

[2] Shortridge D , Gales AC, Streit JM et al Geographic and temporal patterns of antimicrobial resistance in *Pseudomonas aeruginosa* over 20 years from the SENTRY antimicrobial surveillance program, 1997–2016. Open Forum Infect Dis 2019; 6: S63–S8. 10.1093/ofid/ofy343 30895216 PMC6419917

[3] Pirnay JP , Bilocq F, Pot B et al *Pseudomonas aeruginosa* population structure revisited. PLoS One 2009; 4: e7740. 10.1371/journal.pone.0007740 19936230 PMC2777410

[4] Oliver A , Mulet X, Lopez-Causape C et al The increasing threat of *Pseudomonas aeruginosa* high-risk clones. Drug Resist Updat 2015; 21–22: 41–59. 10.1016/j.drup.2015.08.002 26304792

[5] Miyoshi-Akiyama T , Tada T, Ohmagari N et al Emergence and spread of epidemic multidrug-resistant *Pseudomonas aeruginosa*. Genome Biol Evol 2017; 9: 3238–45. 10.1093/gbe/evx243 29202180 PMC5726472

[6] Barrio-Tofino ED . *Pseudomonas aeruginosa* epidemic high-risk clones and their association with horizontally-acquired β-lactamases: 2020 update. Int J Antimicrob Agents 2020; 56: 106196. 10.1016/j.ijantimicag.2020.106196 33045347

[7] Borgianni L , Prandi S, Salden L et al Genetic context and biochemical characterization of the IMP-18 metallo-beta-lactamase identified in a *Pseudomonas aeruginosa* isolate from the United States. Antimicrob Agents Chemother 2011; 55: 140–5. 10.1128/AAC.00858-10 21041509 PMC3019669

[8] Toval F , Guzman-Marte A, Madriz V et al Predominance of carbapenem-resistant *Pseudomonas aeruginosa* isolates carrying blaIMP and blaVIM metallo-β-lactamases in a major hospital in Costa Rica. J Med Microbiol 2015; 64: 37–43. 10.1099/jmm.0.081802-0 25355933

[9] CLSI . Methods for Dilution Antimicrobial Susceptibility Tests for Bacteria That Grow Aerobically—Eleventh Edition: M07. 2018.

[10] CLSI . Performance Standards for Antimicrobial Susceptibility Testing- thirty third *Edition: M100*. 2023.

[11] Magiorakos AP , Srinivasan A, Carey RB et al Multidrug-resistant, extensively drug-resistant and pandrug-resistant bacteria: an international expert proposal for interim standard definitions for acquired resistance. Clin Microbiol Infect 2012; 18: 268–81. 10.1111/j.1469-0691.2011.03570.x 21793988

[12] Molina-Mora J , Garcia F. Molecular determinants of antibiotic resistance in the Costa Rican *Pseudomonas aeruginosa* AG1 by a multi-omics approach: a review of 10 years of study. Phenomics 2021; 1: 129–42. 10.1007/s43657-021-00016-z 35233560 PMC8210740

[13] Chowdhury P , Scott M, Djordjevic S. Genomic islands 1 and 2 carry multiple antibiotic resistance genes in *Pseudomonas aeruginosa* ST235, ST253, ST111 and ST175 and are globally dispersed. J Antimicrob Chemother 2017; 72: 620–2. 10.1093/jac/dkw471 27999026

[14] Mohanam L , Menon T. Coexistence of metallo-beta-lactamase-encoding genes in *Pseudomonas aeruginosa*. Indian J Med Res 2017; 146: S46–52. 10.4103/ijmr.IJMR_29_16 29205195 PMC5735570

[15] Pournaras S , Maniati M, Petinaki E et al Hospital outbreak of multiple clones of *Pseudomonas aeruginosa* carrying the unrelated metallo-beta-lactamase gene variants blaVIM-2 and blaVIM-4. J Antimicrob Chemother 2003; 51: 1409–14. 10.1093/jac/dkg239 12716773

