# Supplementary material for: Endemicity of Pseudomonas aeruginosa producing IMP-18 and/or VIM-2 MBLs from the high-risk clone ST111 in Central America
Source: JAC Antimicrob Resist. 2023 Aug 1;5(4):dlad092. doi: 10.1093/jacamr/dlad092 (PMC10391700; doi:10.1093/jacamr/dlad092)
Supplement: dlad092_Supplementary_Data [file dlad092_supplementary_data.docx]

Supplemental Table 1: Antimicrobial susceptibility of ST111 *Pseudomonas aeruginosa* isolates

| Isolate identifier | PA1 | PA2 | PA3 | PA4 | PA5 |
| --- | --- | --- | --- | --- | --- |
| beta-lactamases detected | IMP-18, OXA-2, VIM-2 | IMP-18, OXA-2, VIM-2 | IMP-18, OXA-2 | VIM-2 | IMP-18, OXA-2, VIM-2 |
| Country | Panama | Panama | Panama | Mexico | Panama |
| Antimicrobial Agent | MIC (mg/L) | | | | |
| Ampicillin-sulbactam | >64 | >64 | >64 | >64 | >64 |
| Aztreonam | 16 | 16 | >16 | 16 | 16 |
| Cefepime | >16 | >16 | >16 | 16 | 256 |
| Ceftazidime | >32 | >32 | >32 | 32 | >32 |
| Ceftazidime-avibactam | >32 | >32 | >32 | 32 | >32 |
| Ceftobiprole | >16 | >16 | >16 | >16 | >16 |
| Ceftolozane-tazobactam | >32 | >32 | >32 | >32 | >32 |
| Ceftriaxone | >8 | >8 | >8 | >8 | >8 |
| Imipenem | >8 | >8 | >8 | >8 | >8 |
| Meropenem | >32 | >32 | >32 | >32 | >32 |
| Piperacillin-tazobactam | 64 | 128 | 128 | 32 | 64 |
| Colistin | 1 | 1 | 1 | 2 | 1 |
| Gentamicin | >16 | >16 | >16 | 8 | 8 |
| Amikacin | >32 | >32 | 8 | >32 | >32 |
| Tobramycin | >16 | >16 | >16 | >16 | >16 |
| Levofloxacin | >16 | >16 | >16 | >16 | >16 |
| Moxifloxacin | >8 | >8 | >8 | >8 | >16 |
| Tetracycline | >16 | >16 | >16 | >16 | >16 |
| Tigecycline | >8 | >8 | >8 | >8 | >8 |
| Trimethoprim-sulfamethoxazole | >8 | >8 | >8 | >8 | >16 |
